# 122 Assessment of Frailty on Discharge Disposition and Long Term Functional Outcomes Following Burn Injury

**DOI:** 10.1093/jbcr/irad045.095

**Published:** 2023-05-15

**Authors:** Vanessa Dellheim, Elizabeth Cuerdon

**Affiliations:** Massachusetts General Hospital, Saugus, Massachusetts; Massachusetts General Hospital, Boston, Massachusetts; Massachusetts General Hospital, Saugus, Massachusetts; Massachusetts General Hospital, Boston, Massachusetts

## Abstract

**Introduction:**

There is an increasing aging population. The goal of our study was to look at how frailty impacts discharge from acute hospitalization as well as long term function at 1 year following their burn.

**Methods:**

After IRB approval, all patients over age 50 who were admitted September 2019-2021 were considered for enrollment. Exclusion criteria included, a non-survival burn and underlying co-morbidities that would affect long term outcomes independent of a burn. Data collected included: age, gender, TBSA, surgical intervention, Canadian Health and Aging Frailty Score (CFS), discharge disposition, Barthel index, and a functional questionnaire. Follow-up phone calls were completed 6 month-1.5 post.

**Results:**

Follow up data obtained via phone call for 30 subjects; the average age was 71 (10.8) years old with 8.6 % (16.5) TBSA, with a CFS average of 2.89 (1.5). 20 (40%) were lost to follow-up. 90 % of patients returned to their baseline and prior living environment. Of the patients who did not return to their prior functional status all had impaired Bartel index prior to their burn injury as well as higher CFS >5, as well as longer LOS (26 day), and were older. Discharge was assessed based on enrollment sample size (n=50), average age 71 yo (10.4), burn size of 7.4% TBSA (13.2), CFS of 3.4 (1.64), and length of stay 20 (25.09) days. 13 patients (26%) were discharge to an IRF, 10 patients (20%) to a SNF, and 27 patients (54%) to home. Patients who discharge home had Lower CFS 2.2, lower age, 69 y.o., and lower TBSA 5.6% compared to patients who were discharged to SNF and IRF. Patients who were discharge to an IRF were older (77yo), had larger TBSA (13.5) and a lower CFS (3) compared to patients who were discharged to a SNF. 80% of patients who discharge to a IRF returned to their prior level of function compared to 40 % who discharged to a SNF

**Conclusions:**

This work helps structure discussions with the geriatric population about expectations to return to their prior level of function. Frailty appears to be associated with outcomes and discharge as lower CFS, older age and larger TBSA were more likely to discharge to IRF and recover to their baseline compared to patients with higher frailty score who were more likely to discharge to a SNF despite younger age and smaller TBSA. This shows that even relatively small burns (8% TBSA) in the elderly can affect ability to care for oneself and ability to go into the community 1 year after their injury.

**Applicability of Research to Practice:**

This can be used by physicians and therapists to aide in discussions about discharge from the hospital as well as long term outcomes expected following their burn injury.

